# Assessing peri-implant bone microarchitecture: conventional vs. osseodensification drilling - ex vivo analysis

**DOI:** 10.1590/0103-6440202405599

**Published:** 2024-03-22

**Authors:** Breno Fortes Bittar, Bruno Salles Sotto-Maior, Karina Lopes Devito, Gustavo Davi Rabelo, Alessandra Silveira Machado, Ricardo Tadeu Lopes, Neuza Maria Souza Picorelli Assis

**Affiliations:** 1 School of Dentistry, Universidade Federal de Juiz de Fora, Juiz de Fora, Minas Gerais, Brazil.; 2 Department of Dentistry, Federal University of Santa Catarina, Florianópolis, Santa Catarina, Brazil.; 3 Nuclear Instrumentation Laboratory - COPPE/UFRJ, Federal University of Rio de Janeiro. Rio de Janeiro, Brazil.

**Keywords:** Osteotomy, X-Ray Microtomography, Dental Implants, Bone and Bones

## Abstract

The aim was to evaluate primary implant stability and bone microarchitecture in two drilling situations, by comparing the conventional technique (CT) and osseodensification (OD) (Versah Burs - Jackson - Mississippi - USA). The implant insertion torque (IT), implant stability quotient (ISQ), and the peri-implant trabecular microstructure were assessed on bone fragments obtained from pig’s tibia (n=12), divided between CT (n=6) and OD (n=6). After the drilling procedure, the implants were installed (3.5x8.5 mm, Epikut - SIN - São Paulo - Brazil). The IT and ISQ were measured using a digital torque wrench and resonance frequency analysis. Then, the bone fragments containing the implants were removed with a trephine and analyzed by Microtomography (µCT, 8.0 µm). The comparison between groups was performed using the unpaired t-test (α=0.05). The results revealed that OD promotes a higher insertion torque (CT: 7.67±2.44 Ncm; OD: 19.78±5.26 Ncm) (p=0.0005), although ISQ was not different (CT: 61.33±4.66; OD: 63.25±4.58) (p=0.48). There was a significant increase in peri-implant bone volume (CT: 23.17±3.39 mm^3^; OD: 32.01±5.75 mm^3^) (p=0.008), and trabecular parameters: separation (CT: 0.4357±0.03 mm; OD: 0.3865±0.04 mm) (p=0.0449), number (CT: 1.626±0.18 1/mm; OD: 1.946±0.13 1/mm) (p=0.007), and thickness (CT: 0.1130±0.009 mm; OD: 0.1328±0.015 mm) (p=0.02). Structure model index (SMI) data demonstrate no significant differences between groups (CT: 1.7±0.2; OD: 1.4±0.4) (p=0.12). In conclusion, OD increases the insertion torque values and promotes beneficial changes regarding bone microarchitecture compared with CT, revealing more peri-implant bone volume with consequent higher primary stability.

## Resumo

O objetivo deste estudo foi avaliar a estabilidade de implante e a microarquitetura óssea em duas técnicas de fresagem, comparando a técnica convencional (CT) e a osseodensificação (OD) (Versah Burs - Jackson - Mississippi - EUA). O torque de inserção do implante (IT), quociente de estabilidade primária (ISQ) e a estrutura trabecular peri-implantar foram avaliados em fragmentos ósseos obtidos de tíbia de porco (n=12), divididos entre CT (n=6) e OD (n=6). Após o procedimento de fresagem, foram instalados implantes (3,5x8,5 mm, Epikut - SIN - São Paulo - Brasil). O IT e o ISQ foram aferidos por meio de um torquimetro digital e análise de frequência de ressonância. Em seguida, os fragmentos ósseos contendo os implantes foram removidos com trefina e analisados ​​por microtomografia computadorizada (µCT, 8,0 µm). A comparação entre os grupos foi realizada por meio do teste-t não-pareado (α=0.05). Os resultados revelaram que a OD promove maior torque de inserção (CT: 7,67 ± 2,44º Ncm; OD: 19,78 ± 5,26 Ncm) (p=0,0005), embora a estabilidade primária não tenha sido diferente (CT: 61.33 ± 4.66; OD:63.25 ± 4.58) (p=0,48). Houve um aumento significativo no volume ósseo peri-implantar (CT: 23,17±3,39 mm^3^; OD: 32,01±5,75 mm^3^) (p=0,0089) e parâmetros trabeculares: separação (CT: 0,4357 ± 0,03 mm; OD: 0,3865 ± 0,04 mm) (p=0,0449), número (CT: 1,626 ± 0,18 1/mm; OD: 1,946 ± 0,13 1/mm) (p=0,007) e espessura (CT: 0,1130 ± 0,009 mm; OD: 0,1328 ± 0,015 mm) (p=0,02) O índice de modelo estrutural (SMI) não demostrou diferença estatisticamente significativa (p=0.1228). Concluindo, OD apresenta maiores valores de torque de inserção e promove mudanças benéficas na microarquitetura óssea em comparação com a TC, revelando maior volume ósseo peri-implantar.

## Introduction

Primary stability is a key factor for a successful treatment with dental implants and is directly related to the bone quantity, density, the implant macro and microgeometry, and the drilling technique ^1^. Aiming to solve the related problems of low primary implant stability (PIS), mostly related to implants installed in regions with low bone density, a new drilling technique was proposed ^2^. The osseodensification was developed by Huwais and Meyer (2017) ^3^ ^4^ ^,^ ^5^ ^,^ ^6^ and the underlying theory of this technique is the use of a novel drill design, which enables the establishment of an environment that enhances primary stability by densifying the walls of the osteotomy site through non-subtractive drilling. The rationale for employing this technique is that densifying the bone that will come into immediate contact with the endosteal device will not only increase primary stability through a stronger physical interlocking between the bone and the implant but also promote faster new bone formation, which could be related due to the osteoblasts of the drilled bone been close to the implant surface ^4^.

New techniques should be investigated properly, and substantial efforts have been made to improve the investigation of the bone and its characteristics evaluating the morphology and other parameters on a sample in a non-destructive manner ^7^. Therefore, the use of high-resolution Microtomography (µCT) to assess trabecular and cortical bone morphology in animal and human models has grown immensely ^8^. In contrast to conventional two-dimensional histological analysis, µCT enables three-dimensional morphometric characterization in a whole volume ^9^. Histomorphometric analysis of bone is considered the gold standard in some situations and has already shown a high correlation between analyses performed on µCT ^10^.

According to the literature regarding the osseodensification technique, it has already demonstrated remarkable outcomes in terms of enhancing both bone-to-implant contact (BIC) and PIS, especially in low-density bones ^5^ ^,^ ^11^. Understanding the diversity of approaches proposed for peri-implant bone augmentation is crucial for achieving satisfactory implant stability. The null hypothesis of this study is that the osseodensification drilling is not different from the conventional technique regarding the trabecular bone microarchitecture and the primary implant stability. To the best of our knowledge, there is no robust scientific literature that has quantitatively evaluated alterations in bone microarchitecture following the osseodensification technique. Thus, this study aimed to evaluate primary implant stability and bone trabecular microarchitecture in two osteotomy drilling situations, by comparing the conventional technique and osseodensification in an ex-vivo model.

## Materials and methods

The present study was conducted in an ex vivo animal model using porcine bones. The animals were not used exclusively for this study and the bones were collected as it was disposable as waste material, so ethical approval was not necessary. Both tibias of a single skeletally mature porcine were collected on the day of the slaughter. To prepare the samples, approximately 15 mm of the articular surface and subchondral bone were removed by transversely sectioning the bones, thereby exposing the medullary portion. This study was performed on 12 samples removed from the tibias, divided into two groups according to the drilling situations: a control group of the conventional technique (CT) (n=6), and a test group of osseodensification (OD) (n=6). No samples were lost during sample processing and analysis. The evaluation of bone microstructure using µCT followed the guidelines proposed by Bouxsein et al. ^8^, which includes the use of terminologies, procedures, images, and reporting of the results. All drilling procedures, mechanical tests, implant installation, and sample preparation were conducted by a single experienced operator.

### Osteotomy and drilling procedures

The tibial bone segment was adapted to a device, so it was kept immobile throughout the whole process. Perforations were made in random locations, respecting the distance of 1 cm from the fragment edges to avoid the medullary region and with approximately 1 cm between each perforation site. The osteotomy sites were determined randomly by the operator, ensuring an unbiased selection process. This approach was employed to mitigate potential confounding factors and to enhance the study's internal validity.

Osteotomy was performed using a contra-angle with 20:1 reduction (Driller - São Paulo - Brazil), under external and constant irrigation with a 0.9% saline solution (Beker - Embu das Artes - Brazil) coupled to an electric motor with electronically controlled rotation, irrigation, and torque (Driller BLM 600 Plus - São Paulo - Brazil).

**Figure 1:**
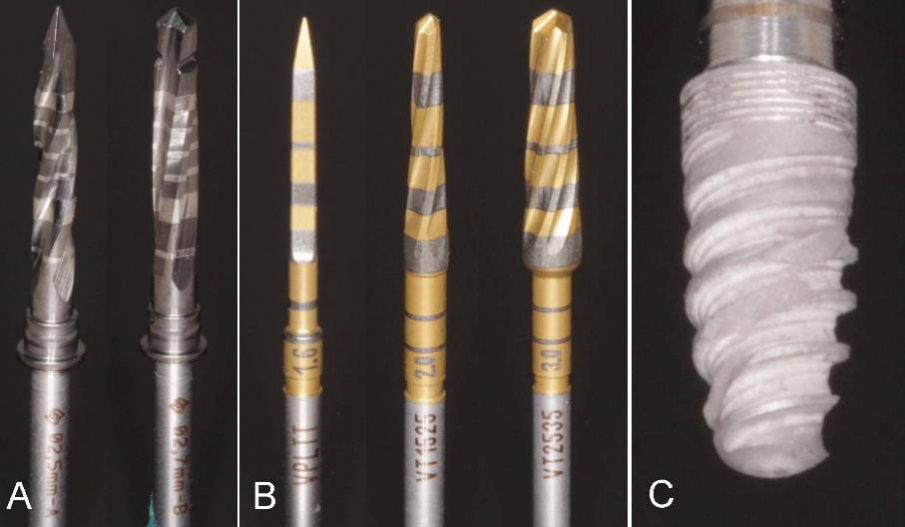
FLI 20 (Ø2.0mm; length 22mm) and FHI 27 (Ø2.7mm; length 22mm) burs (A). VPLTT, VT 1525 (Ø2.5mm; 20mm length) and VT 2535 (Ø3.5mm; 20mm length) burs (B). Epikut Implant 3.5x8.5 S.I.N (C)

**Figure 2:**
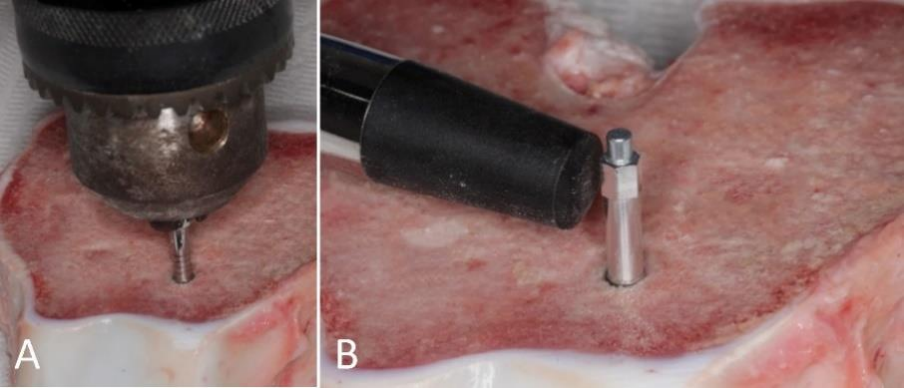
Measurement of insertion torque with a precision digital torque meter (A). Measurement of implant stability with resonance frequency analysis (B).

The osteotomies in the CT group were performed using the FLI 20 burr (SIN - São Paulo - Brazil), followed by the FHI 27 burr (SIN - São Paulo - Brazil) (Figure 1A) with a clockwise rotation of 1200 and 800 rpm, respectively, as per manufacturer guidance (SIN - São Paulo - Brazil) (Figure 2). The OD group had osteotomies initiated with the pilot bur (VPLTT) in a clockwise rotation, followed by VT 1525 (ø2) and VT 2535 (ø3), in counterclockwise direction with a rotation of 1200 rpm, as instructed in the manufacturer guide (Versah Burs - Jackson - Mississippi - USA) (Figure 1B).

Following each perforation, a 3.5x8.5 mm morse taper implant (Epikut - S.I.N Implant System - São Paulo - Brazil) (Figure 1C) was immediately installed at a 1.5 mm intraosseous level using a contra-angle with 20:1 reduction (Driller - São Paulo - Brazil). The implant characteristics were hybrid macrogeometry with thread pitch design, micro threads in the cervical region, and convergent cervical profile, in addition to inverted and double support cutting threads. Its cutting and compressive design makes it suitable for low-density bones.

### Assessment of insertion torque (IT) and primary implant stability (PIS)

The measurement of IT (Ncm) (Figure 2A) of each implant was performed using a precision digital torque meter (Instrutherm TQ-680 - São Paulo - Brazil). The PIS (Figure 2B) was assessed by the Osstell® Mentor (Osstell - Gothenburg - Sweden) with a SmartPeg Type 53 (Osstell - Gothenburg - Sweden) attached to each implant to determine the values of the implant stability quotient (ISQ). An arithmetic mean of the values referring to all the faces of the implant (anterior, posterior, medial, and distal) was obtained ^12^.

### Sample preparation

**Figure 3:**
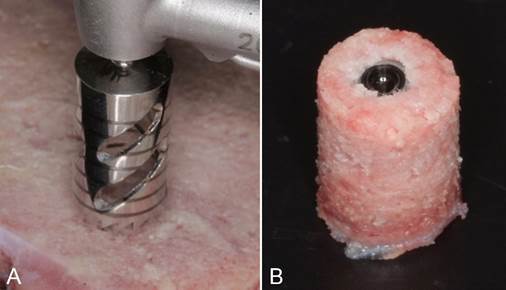
Removal of the bone fragment (sample) containing the implant at its center with a Ø8mm trephine (A). Bone fragment (sample) with the implant at its center (B)

A trephine with 8mm diameter (DSP Biomedical - São Paulo - Brazil) was used to extract a bone fragment containing the implant positioned at its center (Fig 3A). The samples had the standard dimension of 12 mm x 8 mm (Figure 3B). These samples were individually preserved in a hermetically sealed package, immersed in a 10% buffered formaldehyde solution (Indalabor - Dores do Indaiá, Brazil).

### Bone volume and trabecular microarchitecture analysis

The samples were scanned using a Bruker High Energy SkyScan 1273 Microtomography (Bruker - Kontich - Belgium). The following acquisition parameters were used: acceleration voltage of 50 kV; current of 160 µA; 8.0 µm pixel size; 1.0 mm aluminum filter; 2240 x 2240 pixel detector matrix; 0.5º rotation step and 360º total rotation. The average scanning time for each sample was 48 minutes. The NRecon software (1.7.0.4) was used to reconstruct the acquired raw images with the following settings: smoothing: 2; ring artifact reduction: 4; beam hardening: 75%; attenuation coefficient range: 0.00 - 0.16. The images were analyzed with the CT Analyzer software (1.17.7.2).

**Figure 4:**
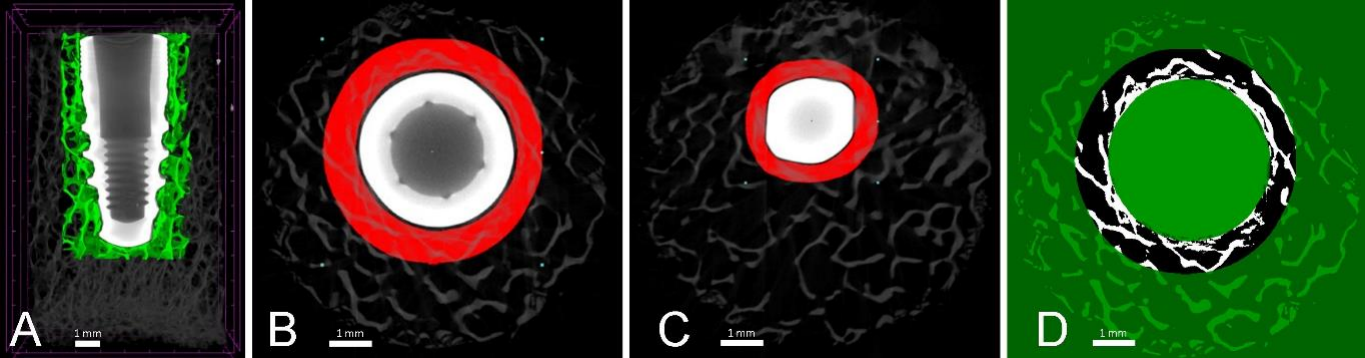
3D graphic representation of the VOI. The green area represents the analyzed volume (A). Axial slice of the VOI (red area), showing the implant, the gap around it, and the analyzed area (B). The axial slice of the VOI shows that due to the irregular implant surface, it needed to be manually adjusted to the implant geometry (C). Axial slide showing the binarized VOI, the green regions were not included in the analysis (D)

A volume of interest (VOI) was defined around the entire body of the implant, comprising approximately 1256 axial slices (Figure4A). The VOI was set to start at 14 pixels (0.112 mm) from the implant's external surface and extended for 1 mm (125 pixels), resulting in a volume of approximately 125 mm^3^ (Figure 4B). According to Garetto et al. ^13^, bone remodeling after implant installation occurs in a region limited to 1 mm around it, and decreases considerably away from this region. The gap between the implant and the beginning of the VOI was included to avoid beam hardening artifacts. Due to the irregular geometry of the implant, the VOI was adjusted manually maintaining its area and gap (Figure 4C).

To analyze the data related to mineralized tissues, image binarization was performed, which converts grayscale pixels into black (void) and white (mineralized tissue). The quantification of mineralized bone tissue was based on the white pixels. The threshold selection for the color histogram of the images was done visually, based on the instructions by Bouxsein et al. ^8^. The selection was based on the similarity to the original non-binarized image, and the threshold range was set from 0 to 35, representing non-mineralized tissue. Above this value, the tissue was considered mineralized (Figure 4D). After binarization, the bone volume (BV), Pore volume (Po.V (tot)), closed porosity (Po.V(cl)), trabecular number (Tb.N), trabecular thickness (Tb.Th), trabecular separation (Tb.Sp), and structure model index (SMI) were analyzed in the whole volume using the 3D Analysis tool. Once the quantitative analysis was completed, the VOI was saved as a 3D model, and representative three-dimensional images were generated for each group using the CTVox software. All these steps in the processing and analysis were performed by one calibrated operator.

### Statistical analysis

The sample size was calculated using G*Power software 3.1.9.6 (Kiel University - Germany). Based on a previous pilot study, a sample size of 6 in each group was calculated considering the homogeneity of the sample, a significance level (α) of 0.05, and a power of 90%, with the main outcome being the bone volume and the related 3D trabecular microarchitecture. Results were expressed as mean ± standard deviation. The data were submitted to Shapiro-Wilk and Grubbs tests, for validation of normality and the presence of outliers, respectively. Comparison between groups was performed using the unpaired t-test. Results were considered statistically significant when p<0.05. The analysis was performed by GraphPad Prism 9 software (La Jolla - California - USA).

## Results

### Insertion torque and primary stability quotient

**Figure 5:**
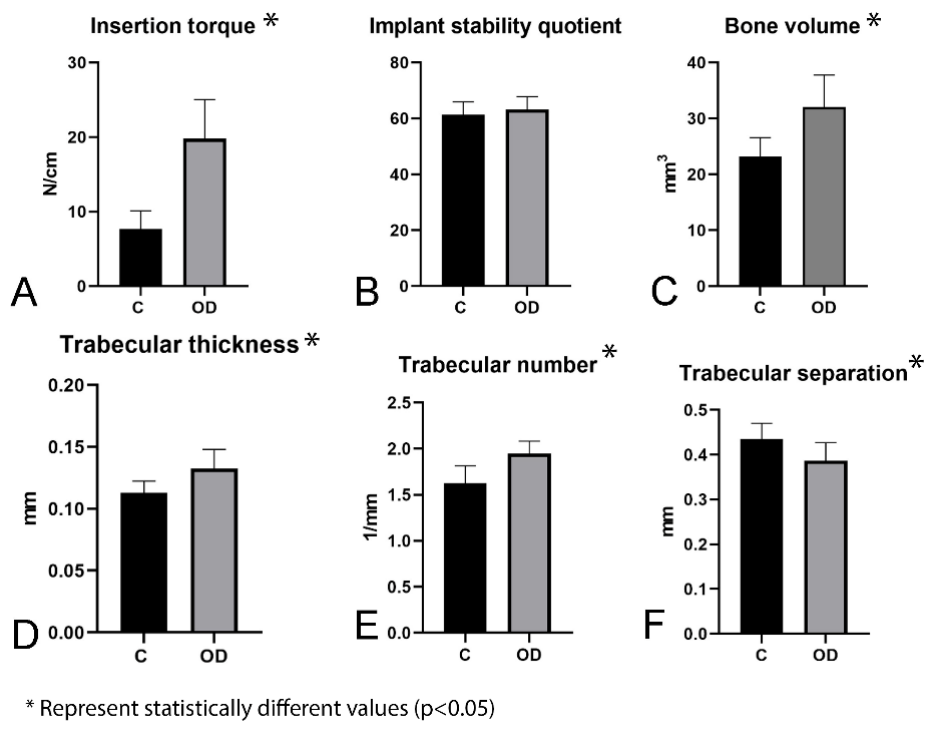
Graph representation of the insertion torque (IT) values (A). Graph representation of the implant stability quotient (ISQ) values (B). Graph representation of the bone volume values (BV) (C). Graph representation of the trabecular thickness (Tb.Th) values (D). Graph representation of the trabecular number (Tb.N) values (E). Graph representation of the trabecular separation (Tb.Sp) values (F).

The OD group had significantly higher values of IT compared to the CT group (CT: 7.67±2.44 Ncm; OD: 19.78±5.26 Ncm) (p=0.0005) (Figure 5A). No statistical differences were found between the groups in terms of ISQ (CT: 61.33±4.66; OD: 63.2 ±4.58) (p=0.48). (Figure5B). Data referring to IT and ISQ are described in Table 1.

**Table 1:** Comparison of the two osteotomy drilling methods: Conventional (CT) versus Osseodensification (OD).

Variable	CT	OD
Implant stability		
IT (N/cm)	7.67 ± 2.44 ^b^	19.78 ± 5.26 ^a^
ISQ	61.33 ± 4.66 ^a^	63.25 ± 4.58 ^a^
Trabecular bone microarchitecture		
BV (mm^3^)	23.17 ± 3.39 ^b^	32.01 ± 5.75 ^a^
BV / TV (%)	18.42 ± 2.68 ^b^	25.47 ± 4.59 ^a^
Po.V (tot)	102.5 ± 3.27 ^a^	94.43 ± 7.16 ^b^
Po.V(cl) (mm^3^)	0.0025 ± 3.27 ^b^	0.0061 ± 7.16 ^a^
Tb.Th (mm)	0.1130 ± 0.009 ^b^	0.1328 ± 0.015 ^a^
Tb.N (1/mm)	1.626 ± 0.18 ^b^	1.946 ± 0.13 ^a^
Tb.Sp (mm)	0.4357 ± 0.03 ^a^	0.3865 ± 0.04 ^b^
SMI (#)	1.776 ± 0.2595 ^a^	1.420 ± 0.4310 ^a^

*Mean ± standard deviation. Equal letters represent statistically equal values. Different letters represent statistically different values.

### Tridimensional trabecular bone microarchitecture

There was a statistically significant difference between groups revealing more peri-implant bone volume in the OD group (CT: 23.17±3.39 mm^3^; OD: 32.01±5.75 mm^3^) (p=0.0089). Also, it demonstrated statistically significant differences regarding the trabecular parameters: Tb.Th (CT: 0.1130±0.009 mm; OD: 0.1328±0.015 mm) (p=0.02), Tb.N (CT: 1.626±0.18 1/mm; OD: 1.946±0.13 1/mm) (p=0.007), and Tb.Sp (CT: 0.4357±0.03 mm; OD: 0.3865±0.04 mm) (p=0.0449). SMI was no different compared to the groups (p=0.99). Data on trabecular parameters are described in Table 1 and in Figure 5 D, E, and F.

## Discussion

Our data revealed that osseodensification drilling resulted in a higher value of insertion torque accompanied by more bone volume and trabeculae number, thicker trabeculae, and lower trabeculae separation values compared with the conventional technique. Therefore, the null hypothesis was rejected as the two osteotomy drilling methods were different. It was already revealed by both ex vivo studies, such as the ones performed by Slete et al. ^14^ and Bandela et al. ^12^, as well as in the vivo study by Lahens et al. ^2^, IT and ISQ are important factors for implant success. Our results pointed to a better condition regarding the site preparation for implant installation when using the osseodensification technique which resulted in higher values of insertion torque. Similarly, in a study by Huwais and Meyer ^15^, higher IT values were observed when implants were installed using the osseodensification technique. It is worth noting that Osstell® equipment was used to assess the ISQ both in the present study and in the study by Huwais and Meyer ^15^. Differently, Almutairi et al. ^3^ also found no differences in the ISQ, although they used Periotest®, which has lower sensitivity than Osstell® ^16^. Additionally, it is important to consider that different animal models were used in the mentioned studies.

Implant thread characteristics such as depth, thickness, and step are crucial in achieving better primary stability ^17^. Studies have shown that wider and deeper threads perform better in micromorphometric and biomechanical aspects than shorter and shallower threads in low-density bones ^18^. The present study used a single implant model with a cutting and compressive design and double threads for inverted support, specifically designed for low-density bones. Therefore, the difference in IT between the groups studied is attributed to the drilling technique used and not to the implant geometry, which is consistent with the clinical findings of Bergamo et al. ^19^ and Lahens et al. ^4^.

According to Pius et al. ^20^, the bone volume is a crucial factor for the success of osseointegration. Osseodensification, being a non-subtractive technique, condenses bone in the surgical site ^21^. This finding is supported by the results of the present study, which showed more peri-implant bone volume compared to the conventional technique. The micromorphometric data suggests that the higher BV value is majorly due to trabecular condensation, as evidenced by the increase in Tb.N and the decrease in Tb.Sp when compared to the conventional technique. In addition, the pore analysis was performed considering the closed spaces within the bone matrix. Once there was a limit on the region of interest, it was clear that the spaces between the trabecular structure were assessed by Tb.Sp, however the spaces inside the trabeculae should be accounted for when assessing the bone volume. In this way, the Po.V (tot) was calculated and was found to be higher in the CT group, suggesting both less volume and less trabecular condensation following the conventional drilling. All these findings are supported by Trisi et al. ^6^, who found an increase in the bone volume percentage (%BV) (approximately 30% higher) for the osseodensification group, although they chose to use conventional histological techniques to quantify these values. In a study conducted by Bhargava et al. ^22^, there were no statistically significant differences in bone volume when using the osseodensification technique, which differs from the findings of the present study. A potential explanation for this divergence may be attributed to the bone used as they have used a porcine rib with 1.5-2mm of cortical bone, which corresponds to a D2 bone. This type of bone is denser than the one used in the present study and may be more resistant to the compression generated by the osseodensification technique, thus not promoting significant changes in bone volume. Summarizing the microarchitectural findings, it can be assumed that osseodensification resulted in different results from the conventional osteotomy drilling technique revealing higher bone volume and trabecular number associated with lower medullar (i.e. trabecular separation) and intraosseous spaces (porous data). One could suppose that for implant stability, more bone and fewer empty spaces (although filled with soft and medullary tissue) would be biomechanically favorable.

The osseodensification technique has been proven to be effective in creating a condensed autograft bone layer around the walls of the osteotomy ^14^ ^,^ ^21^, as supported by the results of the present study. This could be related to the result of lower values in Po.V(tot) in the OD group, together with the higher values for BV. Nonetheless, it is important to understand that this condensed bone layer plays a crucial role in providing immediate mechanical stability to the implant through a stronger physical interlocking between the bone and the implant, which is significant in the context of osseointegration. Additionally, the bone fragments compacted during the osseodensification act as nucleating agents, promoting accelerated osteogenesis within the implant bed, and facilitating the rapid formation of new bone ^19^ ^,^ ^23^.

The structure model index (SMI) is an index developed to assess the plate-like or rod-like nature of the trabecular bone structure. The SMI scale assigns a value of 0 for perfectly plate-like structures, 3 for perfectly rod-like structures, and 4 for perfectly spherical structures ^8^. The relative ratio of rods to plates within trabecular bone is believed to play a significant role in determining bone's mechanical strength, with plates generally considered to possess superior mechanical properties compared to rods ^24^. Based on the findings of this study, the SMI data did not exhibit statistically significant differences. Therefore, it can be inferred that the trabeculae structure in both drilling situations was closer to the plate-like, however not differing comparing both drilling methods.

It is important to consider the limitations of this study, such as in vitro analysis and the use of a single implant model. Further laboratory and clinical studies, maybe including cases associated with bone grafts, are needed to confirm these findings and provide more insight into the tissue responses to the osseodensification technique. Overall, the results of this biomechanical study suggest that osseodensification may be a useful technique for improving primary implant stability in situations where there is little or no cortical bone, such as in immediate implant placement after tooth extractions or cases of intraseptal implantation.

In conclusion, the osseodensification drilling technique revealed higher values of trabecular bone volume, accompanied by a higher number of trabeculae, and reduced medullary and intraosseous spaces compared with the conventional drilling method. In this context, one might posit that osseodensification has the capability to enhance primary stability by increasing insertion torque and that this would be linked to a greater amount of bone available to support the implant.
